# Case Report: Clinical pathological characteristics, diagnosis, and treatment analysis of eosinophilic solid and cystic renal cell carcinoma: experience from a five-case series

**DOI:** 10.3389/fonc.2025.1675819

**Published:** 2025-11-18

**Authors:** Guo Run Zi, Run-lin Feng, Xin Guo, Da-jiang Zhang, Dong-Lin He, Chang-xing Ke

**Affiliations:** 1 Department of Urology, The Second Affiliated Hospital of Kunming Medical University, Kunming, China; 2 Department of Pathology, The Second Affiliated Hospital of Kunming Medical University, Kunming, China

**Keywords:** ESC-RCC, renal cell carcinoma, pathological characteristics, treatment, diagnosis

## Abstract

**Background:**

Eosinophilic solid and cystic renal cell carcinoma (ESC-RCC) is a rare renal tumor subtype. Accurate diagnosis and effective management remain challenging due to its distinct but often overlapping features with other renal tumors. This study aims to characterize the clinicopathological features, management, and outcomes of a five-case series to improve diagnostic and therapeutic strategies for this entity.

**Methods:**

We retrospectively analyzed five patients with pathologically confirmed ESC-RCC from 2020 to 2025. Data on clinical presentation, imaging, histopathology, immunohistochemistry (IHC), surgical management, and long-term follow-up were collected.

**Results:**

The majority of patients (4/5) presented with flank pain. Imaging revealed solitary, well-demarcated cystic–solid masses with progressive enhancement in solid components and a lack of enhancement in cystic areas. Histopathology consistently showed a mixed growth pattern with eosinophilic, hobnail-like cells and distinct perinuclear halos. IHC was crucial for diagnosis, with consistent CK20^+^/CK7^−^/CD117^−^ immunoprofiles. While most patients had a good prognosis with surgical resection, our series also highlighted a young patient (29 years old) and cases with metastatic potential and recurrence.

**Conclusion:**

ESC-RCC exhibits a unique clinicopathological and immunophenotypic profile. Early detection and complete surgical resection are critical for a favorable outcome. The potential for metastasis and recurrence underscores the need for genetic testing (e.g., *TSC* gene mutations) and multidisciplinary collaboration to guide individualized treatment.

## Case report

Between 2020 and 2025, the Second Affiliated Hospital of Kunming Medical University recorded five patients (two men and three women) who were diagnosed with eosinophilic solid and cystic renal cell carcinoma (ESC-RCC) after surgery. The age range was 29–62 years old, and the mean age was 52 years. All patients had single tumors with a diameter of 2.1–12.4 cm. Each patient was subjected to surgical resection, pathological examination, immunohistochemistry, and retrospective imaging analysis. **Table 1** contains a detailed description of the cases.

**Table 1:** Information of the patients.

Case	Age/Gender	Position (kidney)	Size (cm)	Surgical modality	Pathological staging	Prognosis (months)
1	62/M	R	3.9	Partial nephrectomy	Not applicable	No recurrence (51)
2	58/M	L	5.0	Radical nephrectomy	pT3a	Death (26) (died from ESC-RCC)
3	54/F	L	2.1	Partial nephrectomy	Not applicable	No recurrence (29)
4	56/F	L	3.9	Radical nephrectomy	pT3a	No recurrence (24)
5	29/F	L	12.4	Radical nephrectomy	pT3aN1Mx	Recurrence (10)

### Case 1

**Figure 1:**
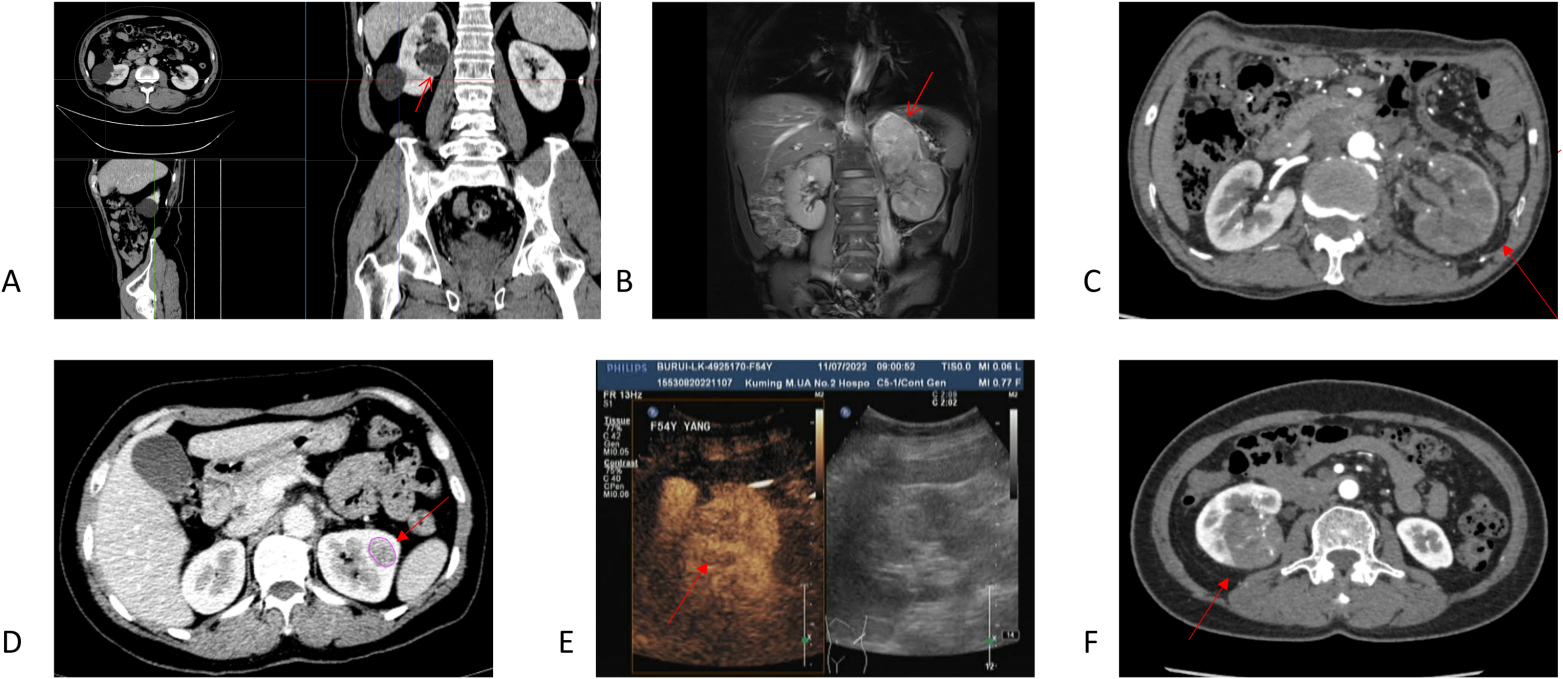
Imaging findings for Cases 1–4 [**(A)**, Case 1; **(B)** and C. Case 2; **(D, E)**, Case 3; F, Case 4]. **(A)** In Case 1, no enhancement was observed in the cystic region post-contrast. MPR view as shown, with arrows indicating the location of the suspected solid mass. **(B, C)** Case 2 demonstrates a T1- and T2-hyperintense mass in the left adrenal region, suggestive of adrenal metastasis. Renal artery CTA reveals no significant enhancement around the cystic lesion with sparse vascularity. **(D, E)** Case 3: A slightly hyperdense nodule with diminished enhancement in both parenchymal and delayed phases. Contrast-enhanced ultrasound imaging: The lesion demonstrated near-parenchymal enhancement in the early phase, with slow resolution in the late phase, appearing slightly hypointense. **(F)** Case 4: Non-contrast CT shows a well-defined tumor margin with a cystic–solid mixture. Contrast enhancement exhibits rapid uptake and washout, revealing punctate calcifications and patchy areas of increased density within the lesion.

A 62-year-old male patient presented with persistent dull discomfort in the right flank without apparent cause 6 years prior, with no other symptoms. Computed tomography (CT) scan revealed multiple renal cysts, the largest measuring 3.9 cm (**Figure 1A**). During surgery, a solid mass adjacent to the renal hilum was discovered alongside the cysts, leading to laparoscopic nephrectomy with cyst decapsulation and partial nephrectomy for the right renal tumor. Postoperative pathology confirmed ESC-RCC. No recurrence was observed during 51 months of follow-up.

### Case 2

A 58-year-old male patient presented with a 1-month history of intermittent, painless gross hematuria and concurrent, dull left flank pain. Both symptoms spontaneously resolved. Initial diagnostic evaluation by CT and magnetic resonance imaging (MRI) revealed a multi-focal tumor involving the left kidney and adrenal gland. A significant tumor thrombus was identified, extending from the left renal vein into the inferior vena cava (IVC), along with concomitant retroperitoneal lymphadenopathy (**Figures 1B, C**). The patient underwent a left radical nephrectomy with IVC thrombectomy, as well as resection of the ipsilateral adrenal gland and proximal ureter. Final histopathological examination confirmed the diagnosis of ESC-RCC, staged as pT3a. Despite surgical intervention, the patient had a progressive disease course and expired 26 months postoperatively.

### Case 3

A 54-year-old man presented with a 1-week history of persistent dull left flank pain and dysuria, following the onset of fever and chills 10 days after a scrub typhus tick bite. A prior MRI, conducted during the treatment for scrub typhus at a municipal hospital, initially identified an intrarenal mass. On admission, subsequent CT and ultrasound examinations confirmed a 2.1-cm solid mass in the left kidney (**Figures 1D, E**). The patient underwent a laparoscopic partial nephrectomy. At a follow-up of 29 months, the patient remained disease-free with no signs of recurrence.

### Case 4

A 56-year-old patient presented with a 1-week history of intermittent, painless gross hematuria and associated right flank pain, both of which resolved spontaneously. Initial CT imaging revealed a 3.9-cm right renal mass. Subsequent enhanced CT findings raised suspicion for malignancy (**Figure 1F**). The patient underwent a laparoscopic radical nephrectomy. Final histopathological examination confirmed the diagnosis of ESC-RCC. The tumor was staged as pT3a, with invasion into the renal pelvis and close proximity to the renal capsule and renal sinus. Postoperatively, the patient was initiated on adjuvant sunitinib therapy; however, the treatment was discontinued due to severe adverse reactions, including grade 3 oral ulcers and thrombocytopenia. At a 24-month follow-up, the patient remained disease-free without recurrence.

### Case 5

**Figure 2:**
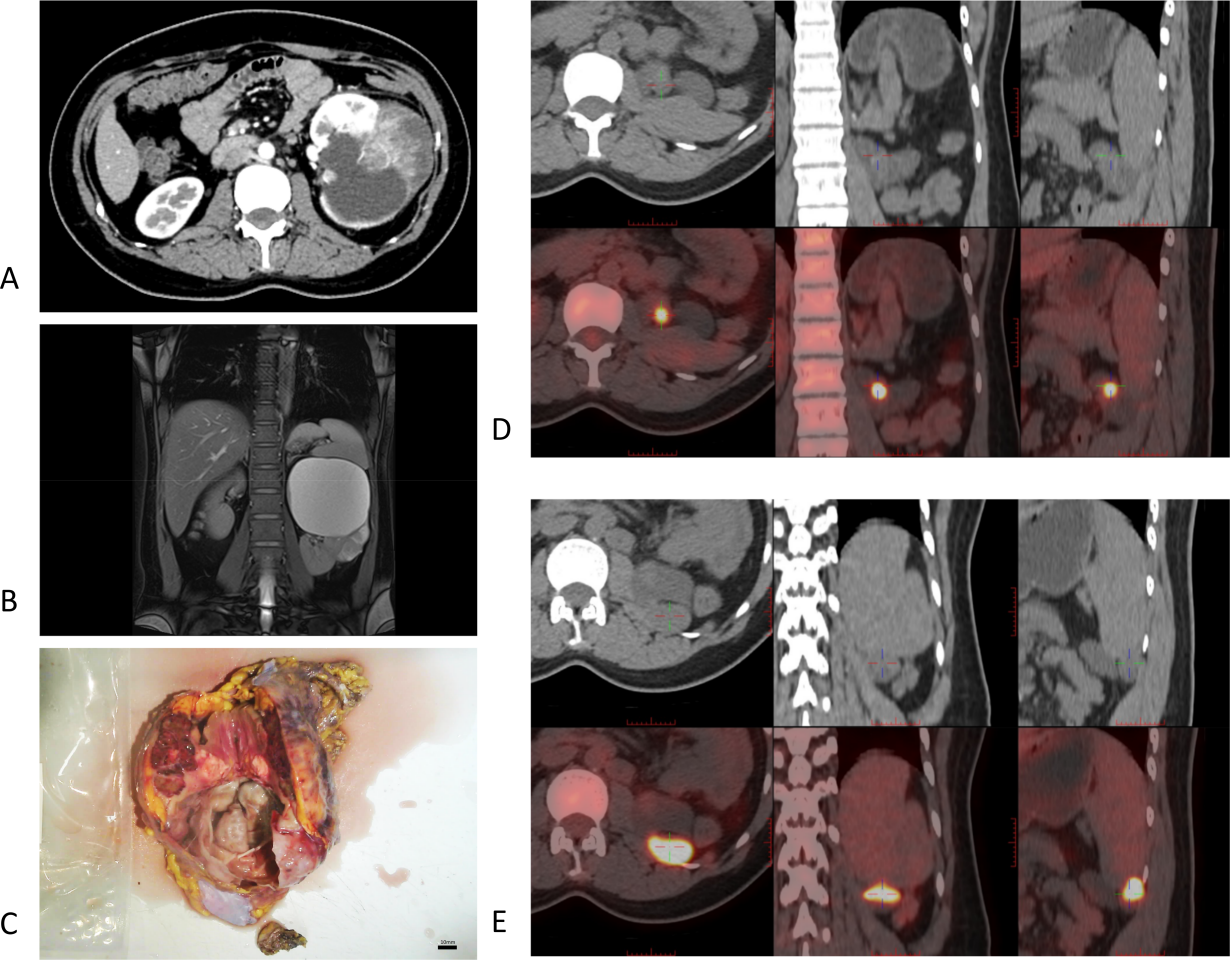
Imaging and gross findings of Case 5. **(A)** The solid components of the tumor showed mild post-contrast enhancement. **(B)** On MRI, the left renal lower pole showed a slightly hypointense signal on T1WI and a slightly hyperintense, heterogeneous signal on T2WI. A prominent T1-weighted contrast-enhanced signal was observed, suggesting possible intratumoral hemorrhage. **(C)** Gross pathology: The kidney was completely dissected with easily separated capsule and fat. The cut surface revealed gray-red to gray-brown masses in the renal pole, with indistinct borders from the surrounding parenchyma. The tumor appeared to invade the renal sinus but not the renal pelvis. **(D, E)** Recurrence on PET-CT: PECT-CT examination showed recurrence: a small amount of fluid accumulation in the left nephrotomy area and multiple occupancy, with increased FDG uptake; the size of the adjacent peritoneum was approximately 2.6 × 1.7 cm, the SUVmax was approximately 10.1, and the size of the renal area after surgery was 4.5 × 3.7 cm, and the SUVmax was approximately 12.

A 29-year-old pregnant woman was initially identified with a large left renal cyst (12.4 × 8.6 cm) during a pre-pregnancy examination 40 days prior. She presented with a complaint of occasional lumbar pain exacerbated by exertion. Enhanced CT revealed a 12-cm non-enhancing cystic lesion in the upper pole of the left kidney and a separate 8-cm solid, heterogeneously enhancing mass in the middle-lower pole. The presence of retroperitoneal lymph node metastases was also noted. The patient underwent a left radical nephrectomy. Final histopathological diagnosis was ESC-RCC, with a pathological stage of pT3aN1Mx. Because of limited hospital resources, genetic testing was not performed. The 5-month postoperative examination showed no signs of recurrence. However, at the 10-month follow-up, tumor recurrence was detected, which was subsequently confirmed by PET-CT (**Figure 2**).

## Discussion

ESC-RCC is an emerging and distinct type of renal tumor. Its formal inclusion as a new entity in the 2022 World Health Organization (WHO) Classification of Urinary and Male Genital Tumors (5th Edition) has since garnered significant attention within pathology and clinical research (1–3). ESC-RCC primarily occurs in women and is characterized by a sporadic (somatic) *TSC* mutation in most cases (4).

Regarding clinical manifestations, although all five of our patients presented with unilateral disease, previous reports of ESC-RCC have shown that it can be multiple and bilateral. In some cases, it may also be associated with other tumors (5). The clinical characteristics of the patients are summarized below:

### Clinical presentation

Four of the five patients were symptomatic upon presentation. The most frequent symptom was flank or lower back pain (4/5), while gross hematuria was reported by a minority of patients (2/5).

### Imaging findings

#### Ultrasound

As a common screening tool, ultrasound examination effectively detected the presence of mixed cystic–solid lesions (**Figure 1E**). CT scan: The tumors were characterized by well-defined borders with mixed cystic and solid components. The cystic regions demonstrated a lack of enhancement post-contrast, whereas the irregular cyst walls and solid components showed heterogeneous enhancement that was typically mild and progressive. These imaging features are consistent with the findings previously reported by Fu et al. and Sandro (**Figures 1A, F**, **2A**) (6, 7).

Some patients’ tumors exhibited rapid contrast enhancement and subsequent washout (**Figure 1F**), a phenomenon that has been previously documented (2). Renal artery computed tomography angiography (CTA) revealed minimal enhancement around the cystic lesions, with an overall sparse vascular distribution (**Figure 1C**). MRI demonstrated a pattern of heterogeneous mixed signals. T1-weighted imaging (T1WI) showed isointense to hypointense areas, while T2-weighted imaging (T2WI) revealed isointense to hyperintense signals. The renal medulla was not prominent and demonstrated a non-uniform enhancement pattern following contrast administration. In Case 2, combined imaging findings strongly suggested a renal tumor with adrenal metastasis, as evidenced by slightly prolonged T1 and T2 signals in the left adrenal region (**Figure 1B**). In Case 5, the MRI scan further characterized the complex nature of the tumor, which presented as a giant upper pole cyst with a distinct solid mass in the middle and lower portions (**Figure 2**). On MRI, the solid component exhibited slightly hypointense signals on T1WI and hyperintense signals on T2WI, with a heterogeneous internal signal pattern. Significant cortical enhancement was noted during T1-weighted contrast-enhanced scanning, which was suggestive of intratumoral hemorrhage.

Building upon an analysis of imaging and pathological features, Fu et al. proposed a three-tiered imaging classification to facilitate the clinical diagnosis of ESC-RCC. This classification system is divided into three types:

- Type 1: Characterized by an equal proportion of cystic and solid components, representing the most frequently observed morphology.
- Type 2: Predominantly cystic, with only a minor solid component.
- Type 3: Predominantly solid.

The solid component of the tumor consistently exhibits distinct imaging features: it presents as isodense or slightly hyperdense on unenhanced CT scans, demonstrates an isointense to slightly hyperintense signal on T1WI, and appears as a low-signal lesion on T2WI. These specific imaging characteristics are in agreement with the CT and MRI findings observed in the cases we report here (8).

In the context of diagnosis, the accurate identification of specific imaging features is paramount for the early detection of ESC-RCC. Consistent with the findings of Fu et al., Yi et al. also highlighted the diagnostic utility of the ratio of cystic to solid components. This has led to the proposed classification of ESC-RCC into three distinct imaging types. Notably, in Type 1 cases, where the cystic and solid components are approximately equal, imaging may reveal a characteristic “lotus-root” pattern. This unique feature can be highly valuable in the differential diagnosis of ESC-RCC from other renal tumor subtypes (9).

### Pathological findings

**Figure 3:**
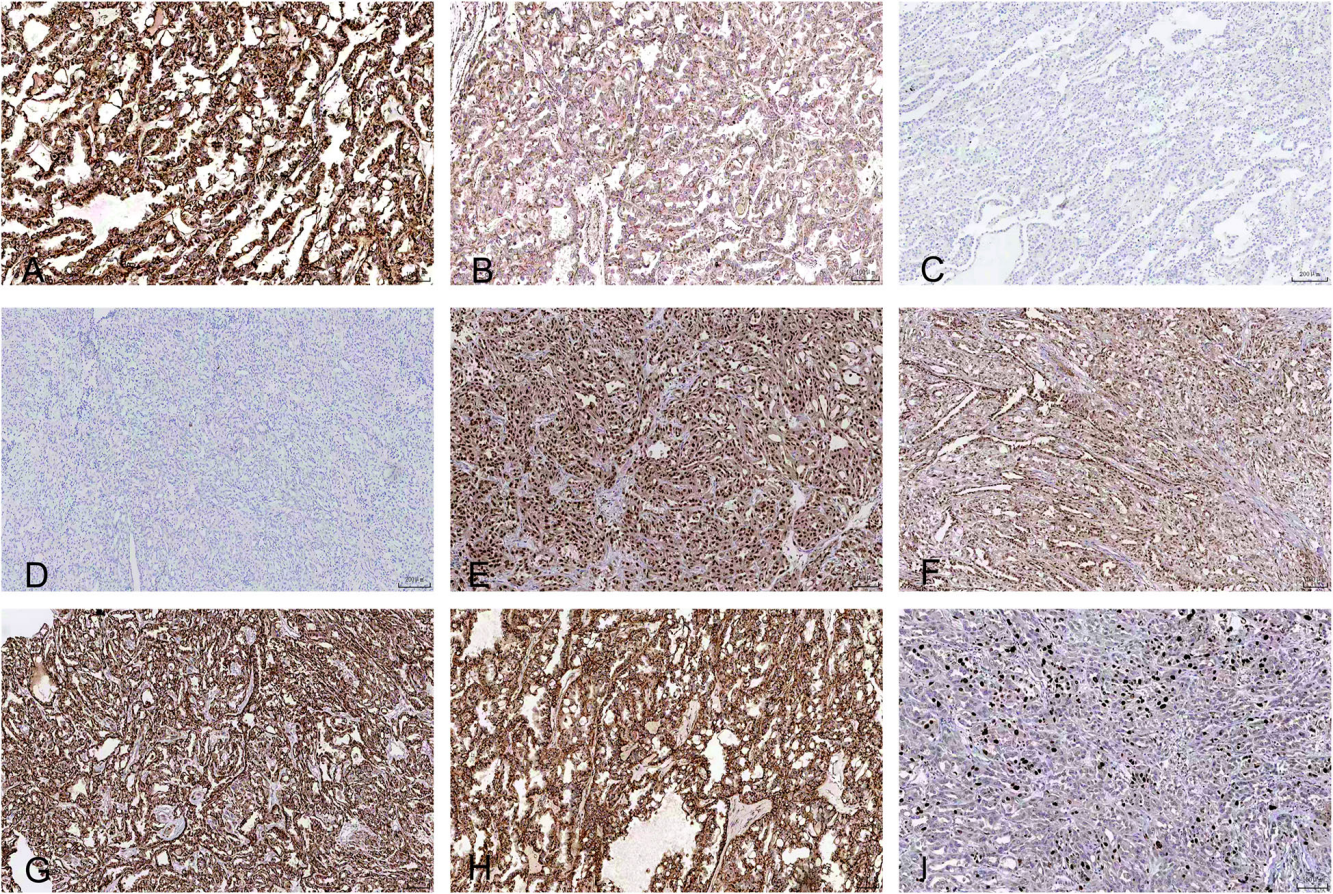
Immunohistochemical features (under the digital pathology slide scanner). **(A)** CK20 shows strong positive expression in the tumor (magnification ×100). **(B)** Ksp-cadherin shows moderate positive expression in the tumor (magnification ×100). **(C)** CD117 shows negative expression in the tumor (magnification ×100). **(D)** CK7 shows negative expression in the tumor (magnification ×200). **(E)** PAX8 shows strong positive expression in the tumor (magnification ×100). **(F)** PAX2 shows strong positive expression in the tumor (magnification ×100). **(G)** P504S shows strong positive expression in the tumor (magnification ×100). **(H)** SDHB shows strong positive expression in the tumor (magnification ×100). **(I)** Ki-67 shows 20% expression in the tumor (magnification ×100).

**Figure 4:**
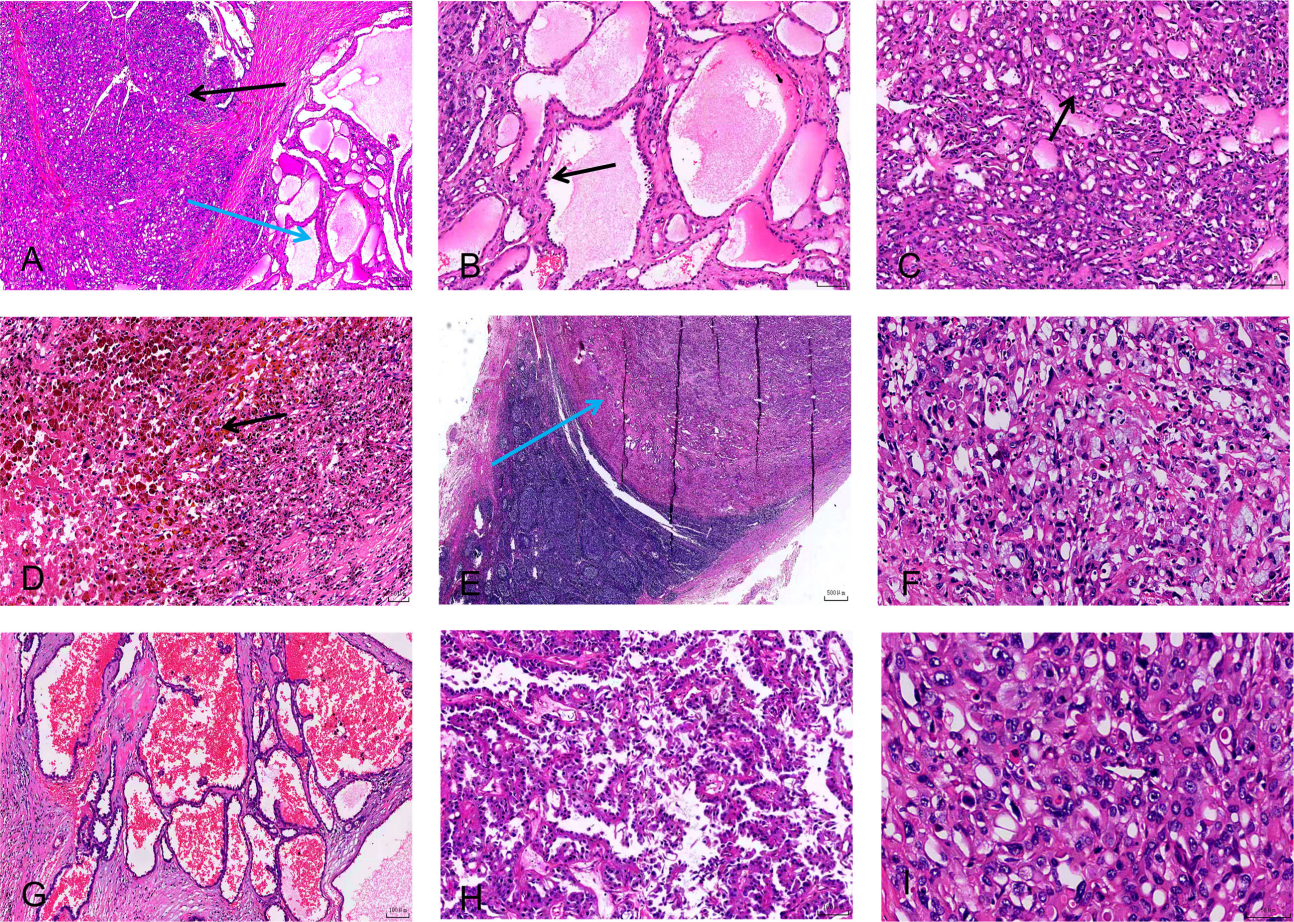
Histopathological features (under the digital pathology slide scanner). **(A)** Low-power field view showing a mixed growth pattern composed of solid structures (black arrow) and cystic structures (blue arrow) (HE stain, magnification ×200). **(B)** The cystic areas comprise cavities of varying sizes lined by eosinophilic, “hobnail” appearance cells (black arrow) (HE stain, magnification ×100). **(C)** The solid region shows diffusely distributed lesions with cells exhibiting acinar or nests of growth and eosinophilic cytoplasm (black arrow) (HE stain, magnification ×100). **(D)** Hemosiderin deposition and areas of necrosis are visible in the surrounding tissue (HE stain, magnification ×50). **(E)** Digital scan section showing metastatic cancer in regional lymph nodes (blue arrow) (HE stain, magnification ×500). **(F)** Mucinous degeneration and foamy cells within the lesion stroma (HE stain, magnification ×50). **(G)** Cystic structures containing copious bloody material (HE stain, magnification ×100). **(H)** Papillary structures within solid components (HE stain, magnification ×100). **(I)** High-power field view showing eosinophilic, vacuolated cells with stippled eosinophilic granules and perinuclear clear margins. Pale purple inclusions are visible in some areas, along with nucleoli and mitotic figures (HE stain, magnification ×50).

The pathological characteristics of ESC-RCCs are key points of research. Pathological examination is the gold standard for the diagnosis of renal tumors. Hartmann et al. (10) described the histological characteristics of ESC-RCC in detail in their study, highlighting its differences from other renal cell carcinomas, such as clear cell renal cell carcinoma (ccRCC). ESC-RCC is generally manifested as a solid cystic structure with clear boundaries (**Figure 2C**). Microscopically (**Figure 4**), the cyst wall is lined by eosinophilic hobnail-shaped cell components, which grow in an alveolar or nest-like manner and have eosinophilic cytoplasm. The tumor cells show abundant eosinophilic cytoplasm, consistent with the pathological characteristics reported in this study (2, 11). In addition, a case of ESC-RCC with melanin deposition by Aldera et al. further expands the morphological spectrum of tumors and suggests that different morphological manifestations should be considered at diagnosis (12). CK20 immunohistochemical positive and CK7 negative are considered as important immunological diagnostic indicators of ESC-RCC. In the reported cases (**Figure 3**) immunohistochemical analysis showed positive results for PAX8 (4/5), P504S (4/5), Vimentin (4/5), SDHB (+) (5/5), ksp-cadherin (5/5), CK20 (5/5), and CD10 (4/5). However, all cases showed negative expression for CD117 (5/5), CK7 (5/5), and CAIX (5/5). The PAX2 portion was partially positive (2/5). The average Ki-67 expression level was 18.6%, consistent with previous reports (13). In this group of four patients, no clinical manifestations or family genetic history of *TSC* was found, and because of the limited hospital medical equipment available, no genetic testing was performed at that time. Pathogenic mutations in the tuberous sclerosis complex (*TSC*)/mTOR signaling pathway are associated with various renal cell tumors, including ESC-RCC. Amir et al. demonstrated that ESC-RCC exhibits loss of the *TSC2* immunoglobulin heavy chain (IHC) and potential pathogenic alterations in the *TSC2* gene (14). The applicability of mTOR inhibitors to *TSC* gene mutation diseases is further elucidated.

### Imaging and pathological data

## Differential diagnosis

Diagnostic challenges: ESC-RCC is easily misdiagnosed as another eosinophilic renal tumor, requiring a combination of morphological triad (solid cystic structure, eosinophilic cytoplasm, and stipple particles), immunophenotype (CK20^+^/CK7^−^), and molecular detection (*TSC* mutation) (15). It is commonly characterized by sporadic mutations in the PI3K pathway, including activation mutations in mammalian rapamycin (mTOR), and/or inactivation alterations in tuberous sclerosis 1 and 2 (*TSC1/2*). However, previous reports showing that these lesions affect patients with tuberous sclerosis (*TSC*) are rare (5, 16).

ESC-RCC needs to be identified from other eosinophilic renal tumors: Chromophobe renal cell carcinoma (eosinophilic subtype): This tumor lacks cystic structures. Tumor cells exhibit a dendritic morphology with thick, transparent cell membranes and a perinuclear halo. They stain pale yellow with colloidal iron staining and are CK7-positive in immunohistochemistry (17). Papillary renal cell carcinoma: Slim papillary structures are common, and the interstitial structure is often accompanied by foam cells or sand granules with low nuclear grading and no acidic granule spots or eosinophilic pellets. Immunolabeled CK7 was positive and CK20 was often negative; furthermore, renal cell carcinoma (PRCC) is only classified into type 1 and type 2 based on morphology, and the specific morphological and immunohistochemical characteristics are needed to distinguish it (18, 19). SDH-deficient renal cell carcinoma: Tumor cells are arranged in a solid arrangement. The tumor cells are usually arranged in a solid pattern and characterized by vacuolated, eosinophilic cytoplasm with flocculent material. CK7 positivity and SDHB negativity are important identification points (20, 21). In addition, it is also necessary to distinguish it from MIT family translocated renal cell carcinoma, oncocytoma, Wilms tumor, renal angiomyolipoma, and acquired cystic disease-associated renal cell carcinoma (ACD-RCC) (2, 22–25).

### Treatment and prognosis

The primary treatments for ESC-RCC are radical nephrectomy (3/5) and partial nephrectomy (2/5). Among five patients followed postoperatively, three achieved tumor-free survival (including one receiving chemotherapy), one died, and one experienced recurrence. In Case 4, the attending physician used sunitinib as systemic chemotherapy, and a similar chemotherapy regimen has been reported. However, the effect varied according to the extent of systemic metastasis of the primary tumor (26). Cases reported by Sakhadeo et al. highlighted manifestations of tumor metastasis (27). In the case study presented here, Case 3 was diagnosed with perirenal, retroperitoneal lymph node, and adrenal metastases at admission, along with venous tumor thrombus formation, indicating a very poor prognosis. Case 5 showed suspected minor lymph node metastases preoperatively but relapsed 10 months postoperatively (**Figure 2**). The patient is currently undergoing outpatient follow-up and plans to visit the oncology department for chemotherapy evaluation. These clinical outcomes underscore the need for more clinical data to better understand its biological behavior.

## Conclusion

ESC-RCC is a rare renal cancer subtype with distinct clinicopathological characteristics. Its definitive diagnosis necessitates a comprehensive analysis of tumor morphology, immunohistochemistry, and molecular profiles. Specifically, the detection of *TSC* gene mutations is pivotal for confirming the diagnosis and provides a theoretical basis for the potential application of mTOR inhibitors. While ESC-RCC is typically observed in middle-aged patients, our report of a 29-year-old woman highlights the possibility of diagnosis in younger individuals. This may be attributed to the lack of early, specific symptoms, leading to delayed tumor detection.

Our case series demonstrates that the biological behavior and prognosis of ESC-RCC are highly dependent on early detection and the achievement of complete surgical resection. A majority of our patients showed a good prognosis even without adjuvant systemic therapy. However, we acknowledge that the small sample size limits our ability to evaluate the efficacy of targeted therapies for advanced disease. Despite its generally indolent nature, existing data indicate that ESC-RCC can be both invasive and lethal in some cases.

Ultimately, multidisciplinary collaboration among pathologists, radiologists, and geneticists is essential to improve diagnostic accuracy and provide a foundation for personalized treatment strategies. As more cases are accumulated and studied, we anticipate a clearer understanding of the tumor’s biological nature and behavior, paving the way for more targeted and effective therapeutic interventions in the future.

## Data Availability

The original contributions presented in the study are included in the article/supplementary material. Further inquiries can be directed to the corresponding authors.
