# Correction: Case Report: Clinical pathological characteristics, diagnosis, and treatment analysis of eosinophilic solid and cystic renal cell carcinoma: experience from a five-case series

**DOI:** 10.3389/fonc.2025.1764820

**Published:** 2025-12-19

**Authors:** Frontiers Production Office

**Affiliations:** Frontiers Media SA, Lausanne, Switzerland

**Keywords:** ESC-RCC, renal cell carcinoma, pathological characteristics, treatment, diagnosis

Reviewer, Junjie Li’s affiliation was erroneously given as “Peking University, China”. The correct affiliation for Junjie Li is “Beijing Hospital, National Center of Gerontology, China”.

The original version of this article has been updated.

